# Supplementary material for: The HIV co-receptor CCR5 regulates osteoclast function
Source: Nat Commun. 2017 Dec 20;8:2226. doi: 10.1038/s41467-017-02368-5 (PMC5738403; doi:10.1038/s41467-017-02368-5)
Supplement: Supplementary file 1 — Supplementary Information [file 41467_2017_2368_MOESM1_ESM.pdf]

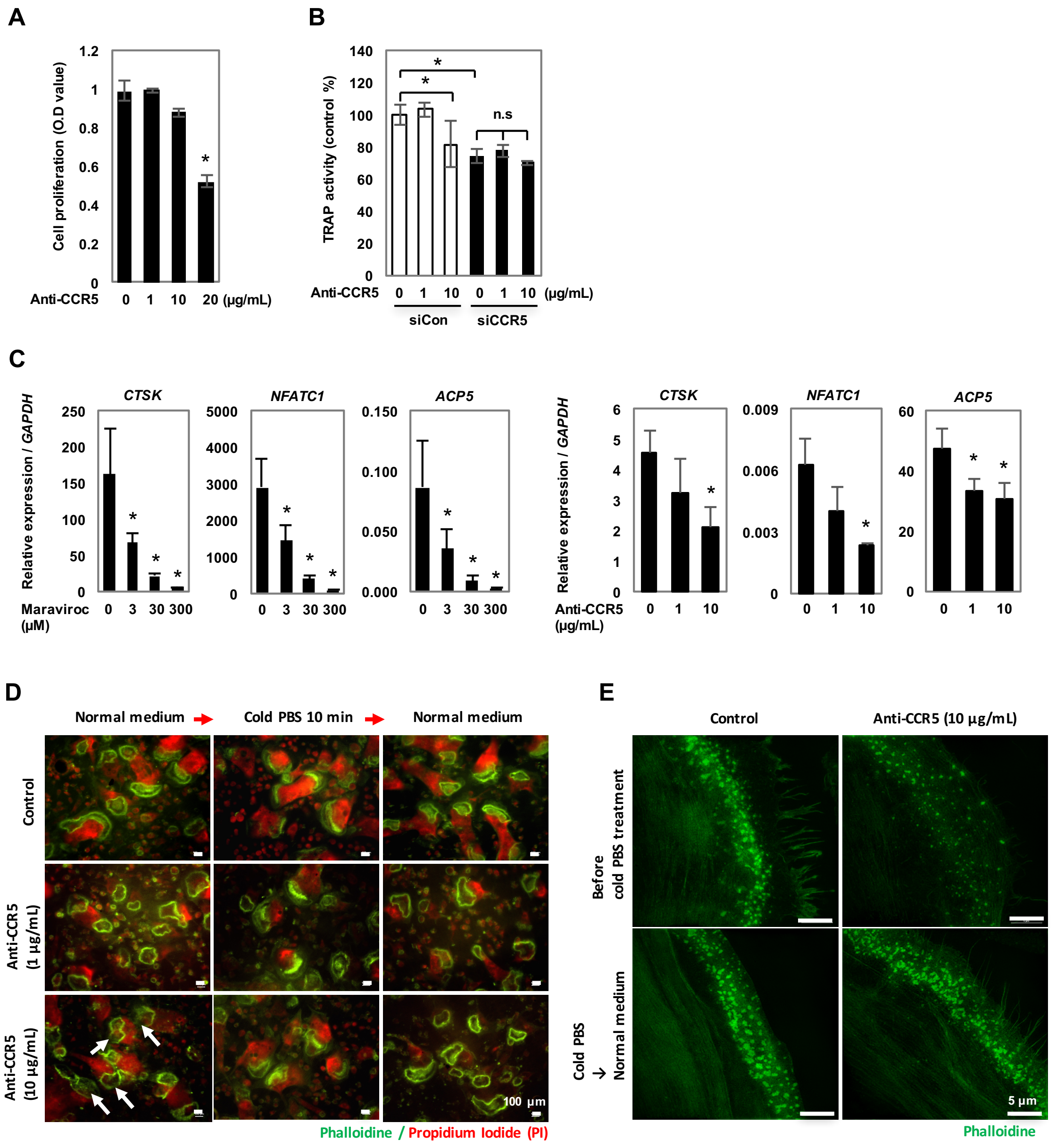

Supplementary Figure 1. The blockade of CCR5 in cultured human osteoclasts. A) Cellular proliferation was measured on human osteoclasts. Cells were cultured in media supplemented with M-CSF but without RANKL. The cells were treated with anti-CCR5 neutralizing antibodies for 3 days (n=5). B) The specificity of the anti-CCR5 neutralizing antibody was examined. U-937 cells, a human monocyte cell line, were transfected with human siCCR5 and control siRNA, and then differentiated with TPA/1,25$(OH)_2D_3$ to become osteoclast-like cells. After transfection, the cells were treated with an anti-CCR5 neutralizing antibody at the indicated concentrations (n=5). C) Osteoclast markers were examined by a real-time Q-PCR and statistically compared (mean ± SD, n=5). U-937 cells were differentiated with TPA/1,25$(OH)_2D_{3,}$ and treated with Maraviroc or

anti-CCR5 neutralizing antibodies at the indicated concentration for 3 days. D) The depletion of the blockade of CCR5 in mature human osteoclasts restored their ability to assemble actin rings. Mature osteoclasts cultured on dentine slices were treated with anti-CCR5 neutralizing antibodies at the indicated concentrations or control antibodies. After exposure to ice cold PBS for 10 min, the cells were cultured in fresh warm medium containing M-CSF and RANKL. The fixed cells were stained with phalloidin-AlexaFluor 488 (in green) and PI (in red) to examine the actin ring reassembly (scale bars, 100 μm, n=5). Arrows indicates incompletely encircled actin rings. E) The reassembly of the actin ring formation of human osteoclasts that had previously been treated with anti-CCR5 neutralizing antibodies was confirmed by SIM imaging. Mature osteoclasts were cultured on a glass-bottomed dish, and were subjected to the same cold shock experiment as described in D. Fixed cells were stained with phalloidin-AlexaFluor 488 (in green). Images were taken by SIM (scale bars, 5 μm, n=5). *$P < 0.05$ by Student's *t*-test. All data are shown as the mean ± SD.

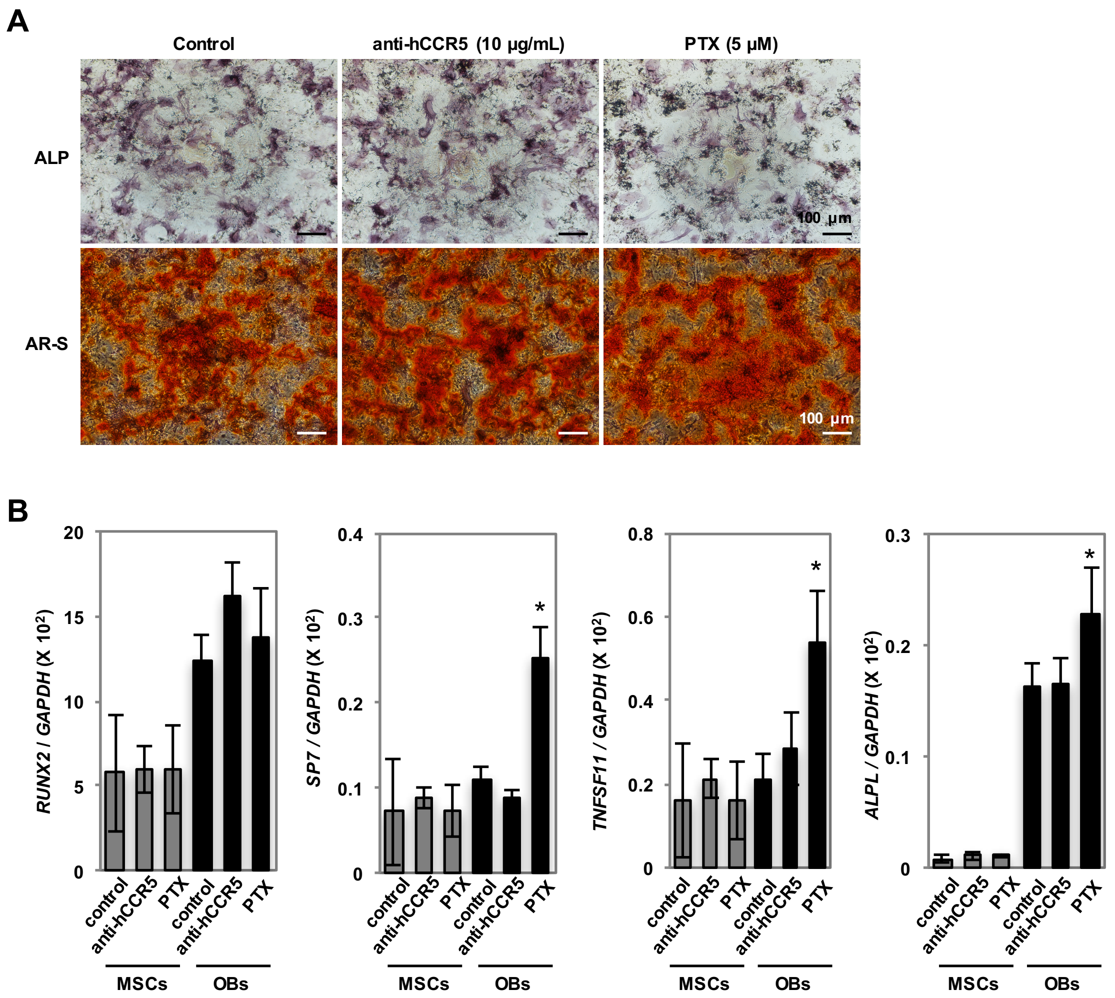

Supplementary Figure 2. The blockade effects of CCR5 on human osteoblast differentiation. A) Human mesenchymal stromal cells (hMSCs) were cultured with osteogenic medium with or without anti-hCCR5 neuAbs for 2 weeks. ALP (upper panels) and Alizarin red (lower panels) staining was performed (scale bars, 100 μm, n=5). B) The relative expression levels of osteoblastic markers such as *RUNX2, SP7/OSTERIX, TNFSF11/RANKL* and *ALPL/ALP* were measured by a real-time Q-PCR, and statistically compared (mean ± SD, n=5). **P* < 0.05 by Student's *t*-test.

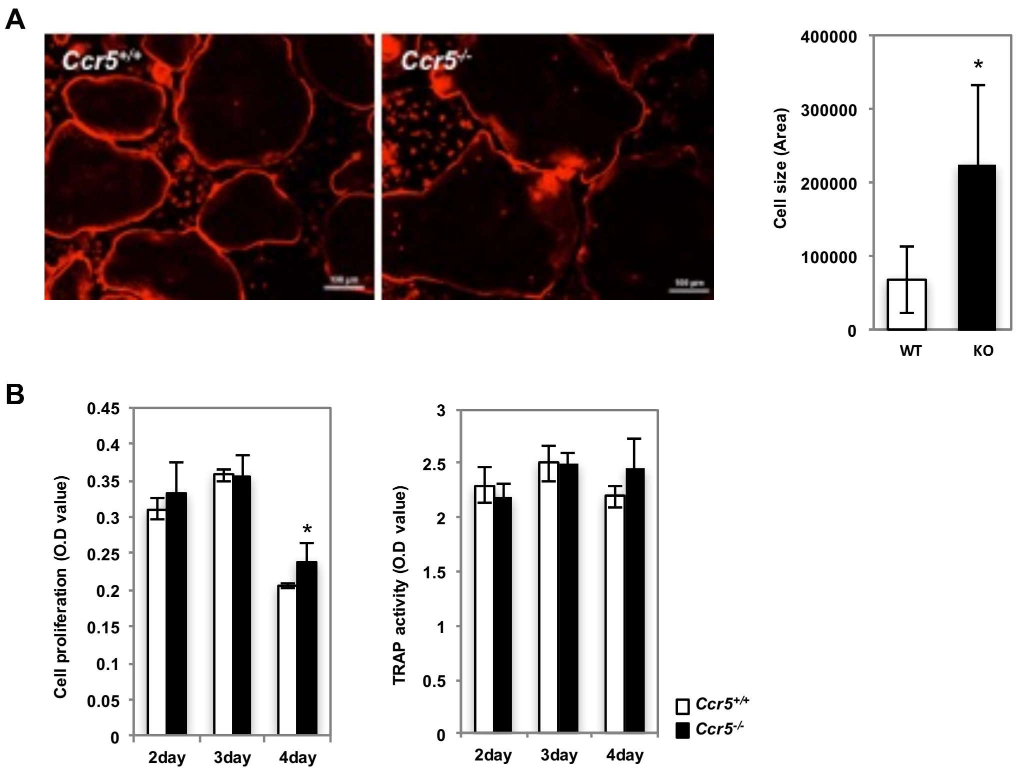

Supplementary Figure 3. *Ccr5*-deficient osteoclasts were larger in size. A) Multinucleated osteoclasts were visualized by staining F-actin with phalloidin-AlexaFluor 568 (in red, scale bars, 100 μm, n=5). The cell size was determined using the ImageJ software program (n=10). B) The proliferation of bone marrow cells of both genotypes cultured with M-CSF for indicated days were assayed (left graph, mean ± SD, n=5). TRAP activity of cells cultured with RANKL for indicated days were measured (right graph, mean ± SD, n=5). **P* < 0.05 by Student's *t*-test.

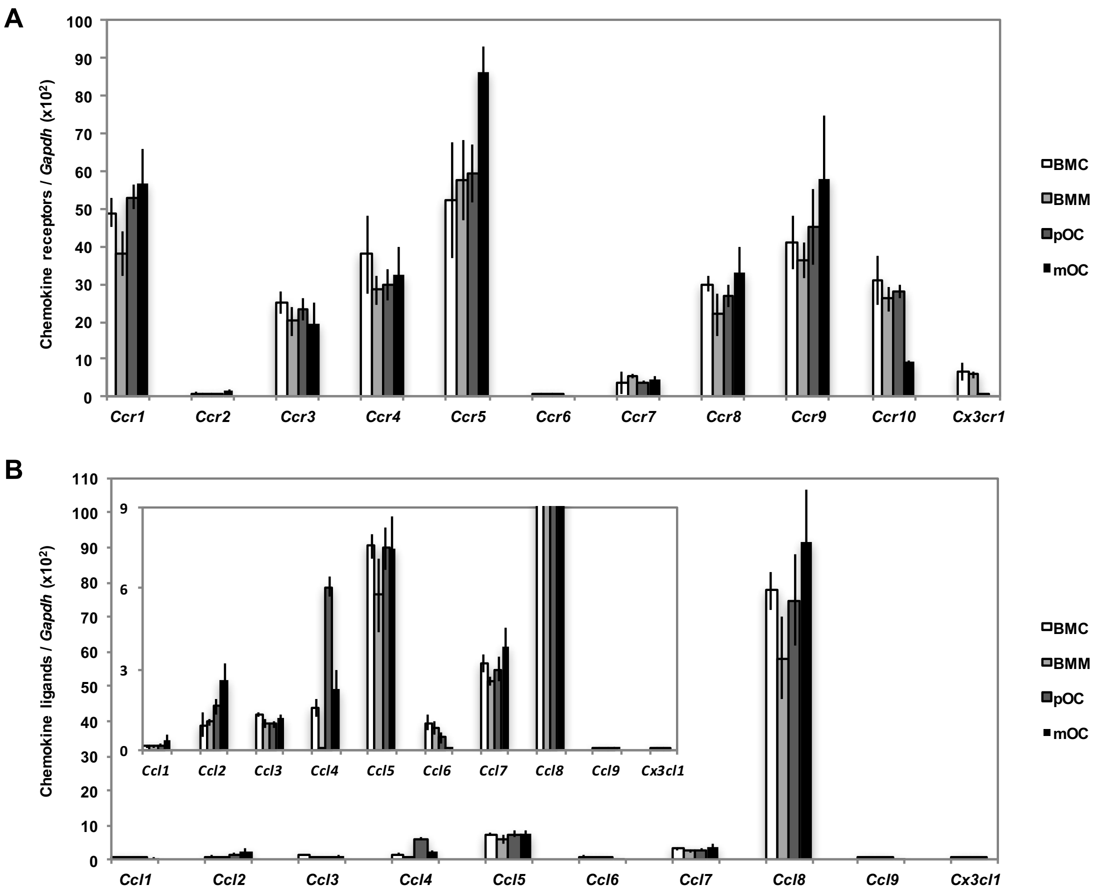

Supplementary Figure 4. The temporal expression of C-C chemokine receptors and C-C chemokines. A, B) The relative mRNA expression levels of mouse CC-chemokine receptors and ligands during osteoclastogenesis were measured by a real-time Q-PCR (mean ± SD, n=5).

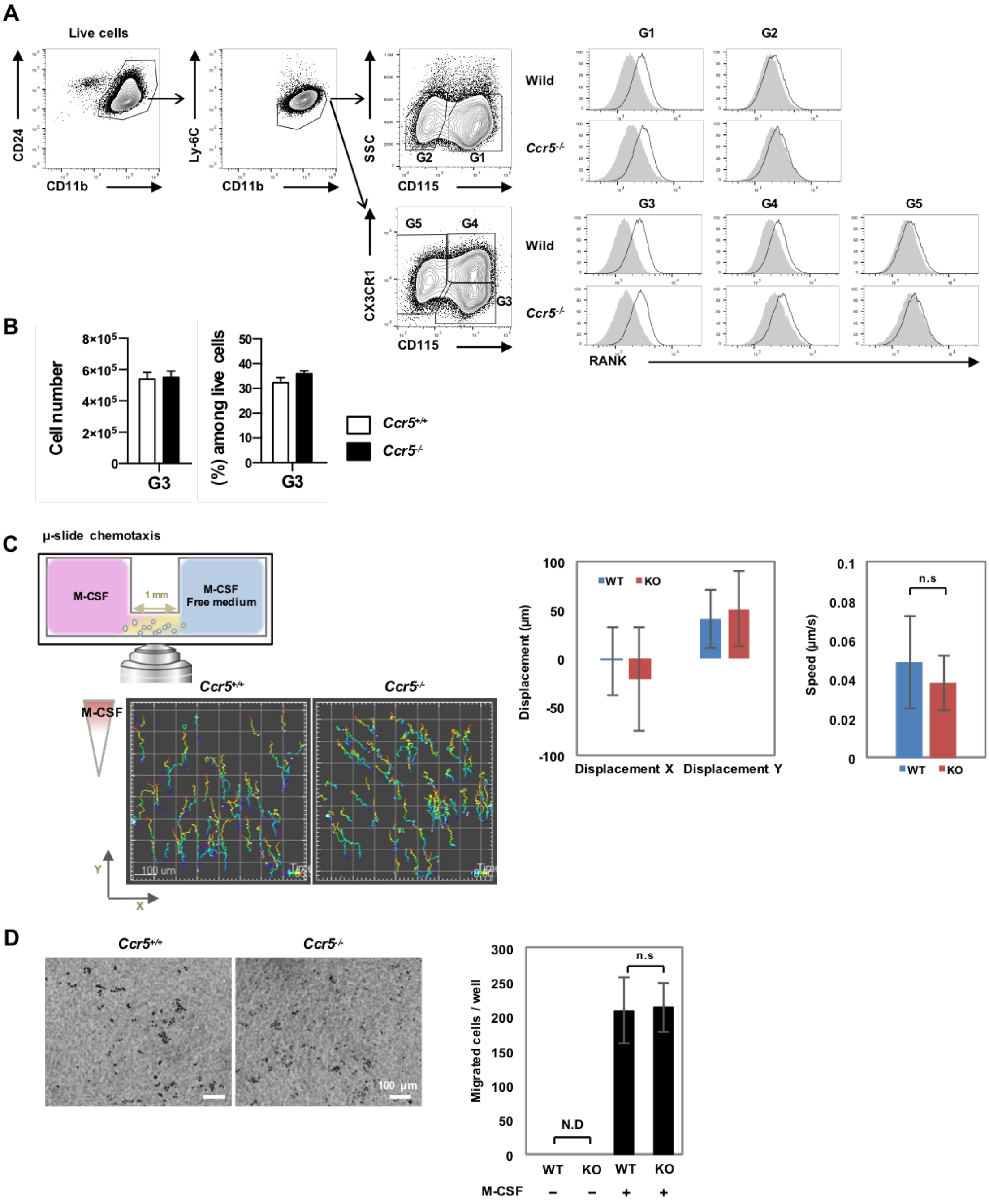

Supplementary Figure 5. Chemotactic and population analyses of *Ccr5*-deficiencent osteoclast precursors. A, B) A population of RANK-positive osteoclastic precursors in CD24^{lo} Ly-6C^{lo} CD11b^{+} CD115^{+} isolated from the bone marrow of wild-type and *Ccr5*-deficient mice after 2 days of culturing with RANKL was analyzed by flow cytometry. Histograms were gated in the CD115^{hi}CX3CR1^{lo} (R3-gated), CD115^{hi}CX3CR1^{hi} (R4-gated), and CD115^{lo}CX3CR1^{+} (R5-gated) subpopulations. The surface expression levels of RANK (bold line) are overlaid on cells stained

with subclass-matched control IgG (shaded histogram). The R3-gated subpopulation contained RANK-positive osteoclastic precursors; thus, this was statistically analyzed. Plots and histograms show representative data from four independent experiments. C) The chemotactic activity of bone marrow cells isolated from wild-type and *Ccr5*-deficient bones were analyzed by time-lapse imaging. M-CSF-induced chemotaxis was examined using μ-slide (shown in a schematic drawing, scale bars, 100 μm, n=3). The movement of osteoclast precursors was monitored with a time-lapse microscopy system. The locomotion (displacement X and Y) and migration velocity were statistically analyzed using the IMARIS software program. D) The cells that had migrated toward M-CSF-containing media through trans-well filters were counted. After 24 hours in culture, the cells were fixed and stained for counting and statistical comparison (scale bar, 100 μm, mean ± SD, n=3). *P < 0.05 by Student's *t*-test.

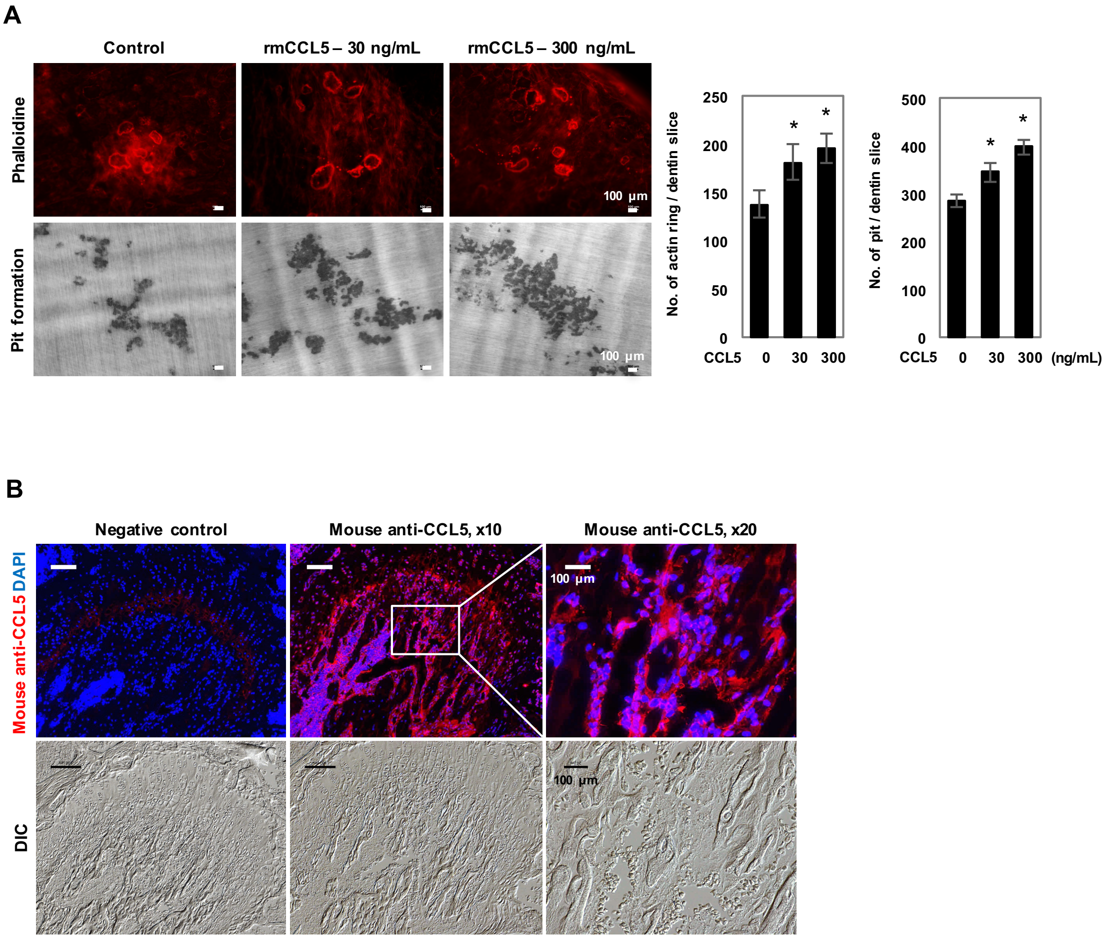

Supplementary Figure 6. CCL5 inoculation enhances the function of mouse osteoclasts. A) The osteoclast function was determined based on actin ring formation and pit formation and the numbers of actin rings and pits were counted. The data are shown as the mean ± SD, n=4 (scale bars, 100 μm). *P < 0.05 by Student's *t*-test. B) Longitudinal tissue sections of femur metaphysis obtained from 8-week-old mice were subjected to immunofluorescence staining using anti-CCL5 (in red) and were stained with DAPI for the visualization of the nuclei. Mouse IgG was used as a negative control. Differential interference contrast (DIC) images corresponding to each fluorescence image are also shown for a morphological comparison (scale bars, 100 μm, n=3).

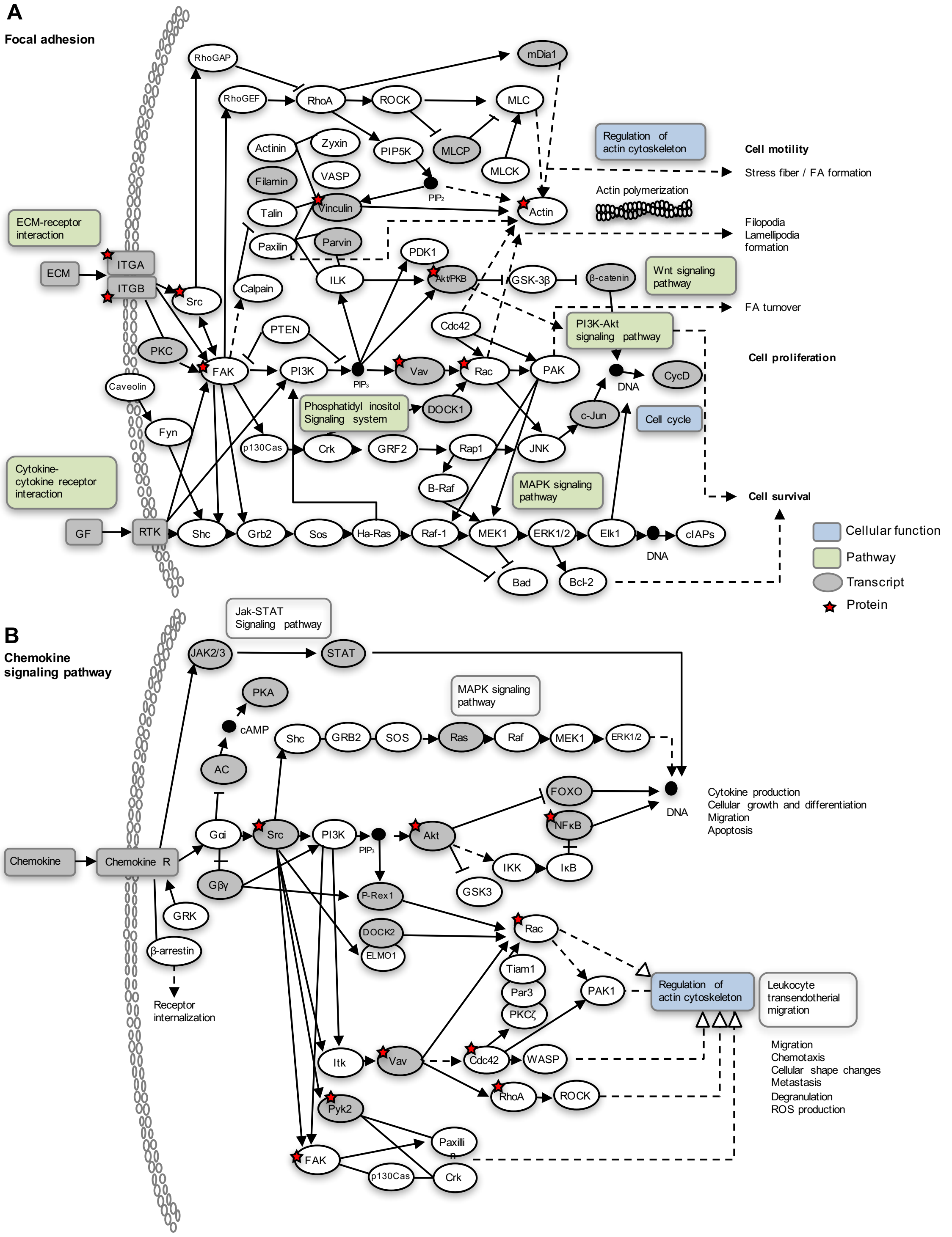

Supplementary Figure 7. CCL5 transcriptionally upregulates the adhesion- and chemokine-mediated pathway. Cultured osteoclasts at pOC stage (see Figure 2A) were incubated with or without recombinant CCL5 for 48 h, and then subjected for RNA sequencing. A, B) The genes that were significantly upregulated by CCL5 in the presence of RANKL were mapped to the cell adhesion and chemokine signaling pathways using the KEGG pathway system. Gray color

indicates the genes that were upregulated by CCL5, while asterisks indicate the proteins that were upregulated and/or phosphorylated by CCL5. The significantly upregulated cellular functions and signaling pathways are indicated by blue and green boxes, respectively.

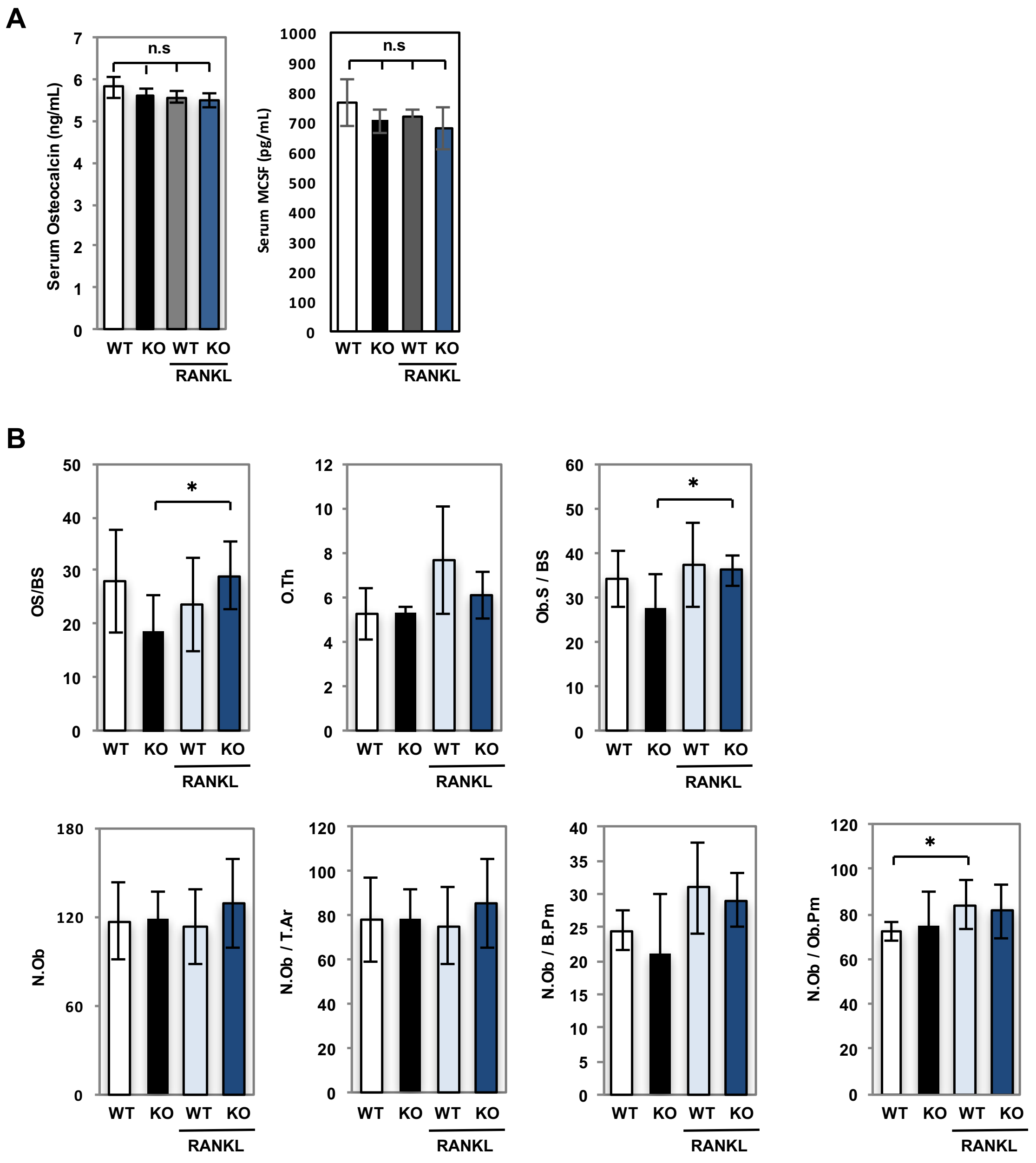

Supplementary Figure 8. Bone histomorphometric analyses of the bones of *Ccr5*$^{-/-}$ mice and their wild-type littermates. A) The levels of mouse serum osteocalcin and M-CSF were measured by an ELISA. *P < 0.05 (by one-way ANOVA) in comparison to WT or RANKL-injected WT. All values are shown as the mean ± SD, n=4-6. B) The osteoid surface per bone surface (OS/BS), osteoid thickness (O. Th), osteoblast surface per bone surface (Ob.S/BS), osteoblast number (N. Ob), osteoblast number per tissue area (No. Ob/T. Ar), osteoblast number per bone perimeter (N. Ob/B. Pm) and osteoblast number per osteoblast perimeter (N. Ob/Ob. Pm) were determined and statistically compared. Each sample was duplicated.

A

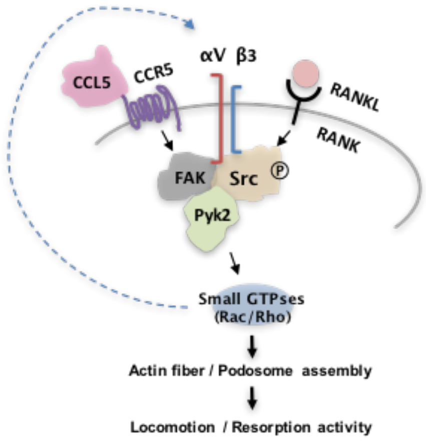

B

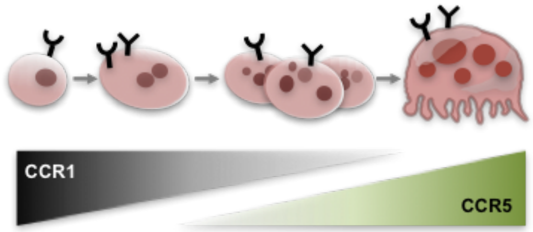

Supplementary Figure 9. Schematized summary of this study. A) Collaboration of CCR5- and RANKL-induced cellular pathways in functional regulation of osteoclasts. CCR5-induced signal sustains the RANKL-induced pathways, which is required to maintain the expression of the molecular components involved in the canonical signaling pathway such as FAK, Src, Pyk2 and Rac, Rho small GTPases that regulates the functional architecture of osteoclasts. The activation of these small GTPases, in turn, sustains *α*Vβ3 integrin-mediated signaling. B) Functional relay of two related-chemokine receptors during osteoclast differentiation. CCR1 is mainly required for the initial differentiation while CCR5 is predominantly essential for later functional differentiation, even though they possibly share the same ligands.

Figure 3C

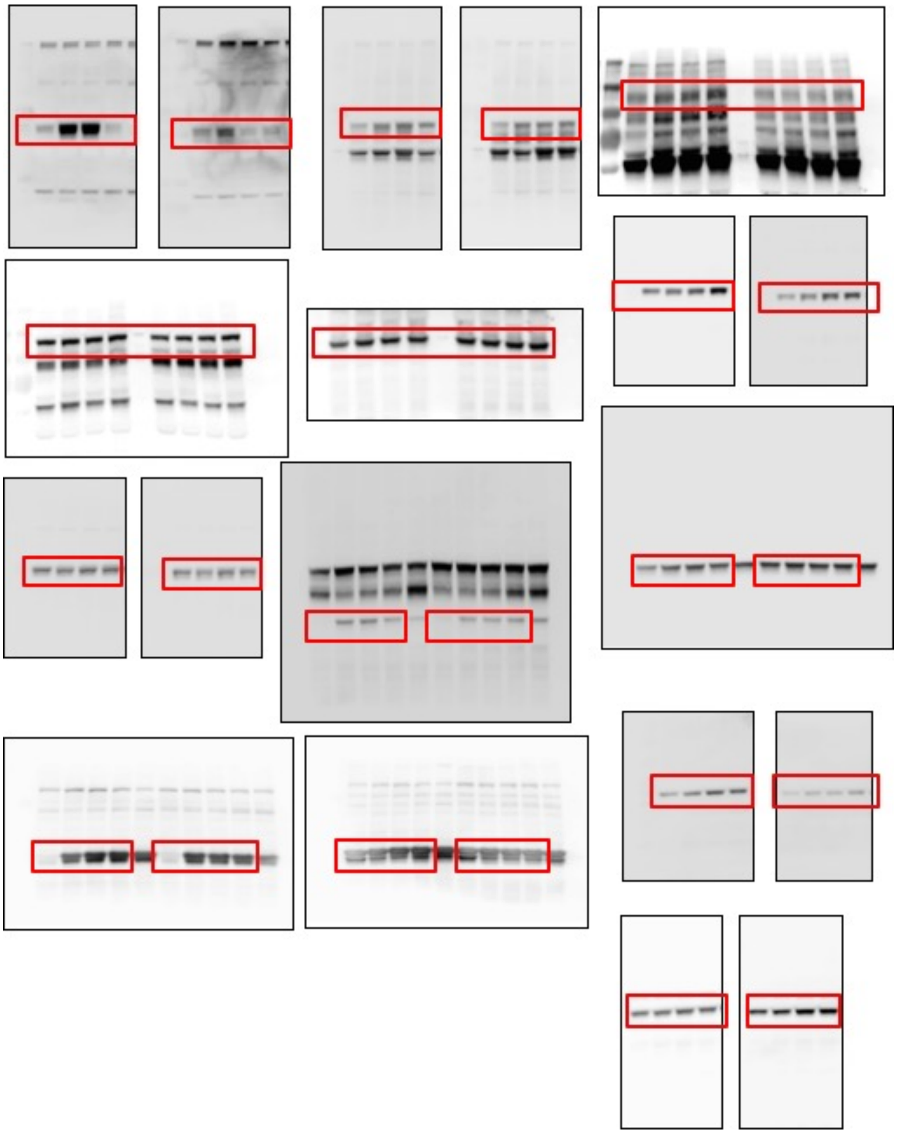

Figure 3D

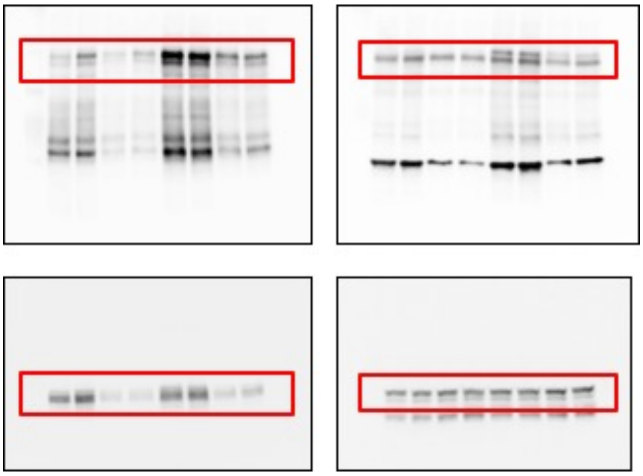

Supplementary Figure 10. Uncropped western blot images. The red sections mark blot and gel results shown in the indicated figures.

Figure 4E

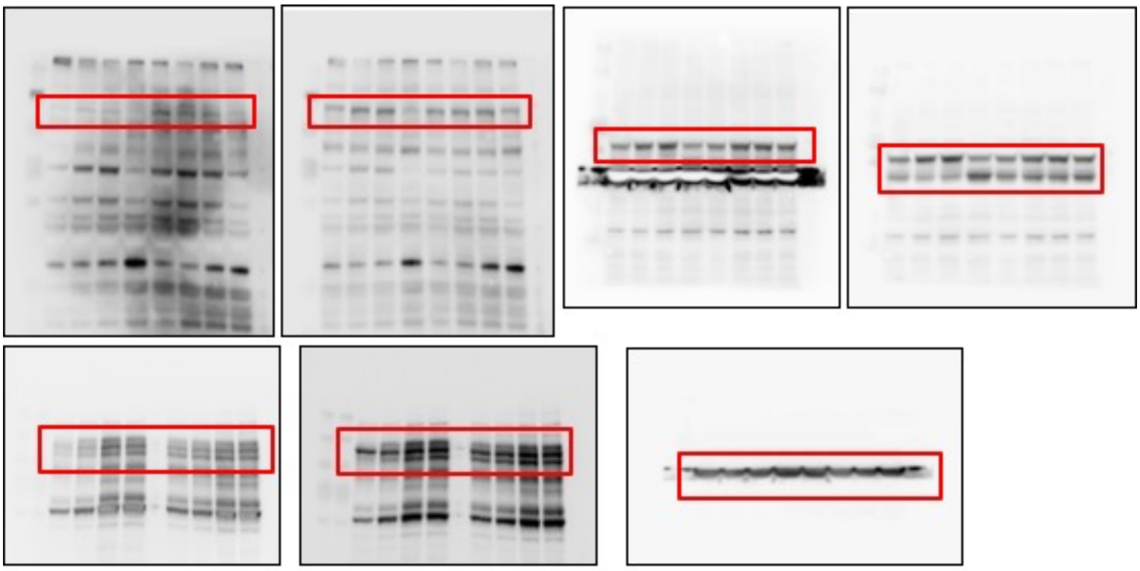

Figure 4F

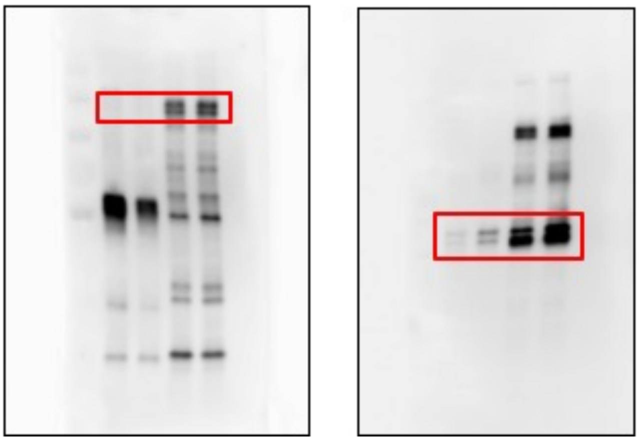

Supplementary Figure 11. Uncropped western blot images. The red sections mark blot and gel results shown in the indicated figures.

Figure 5A

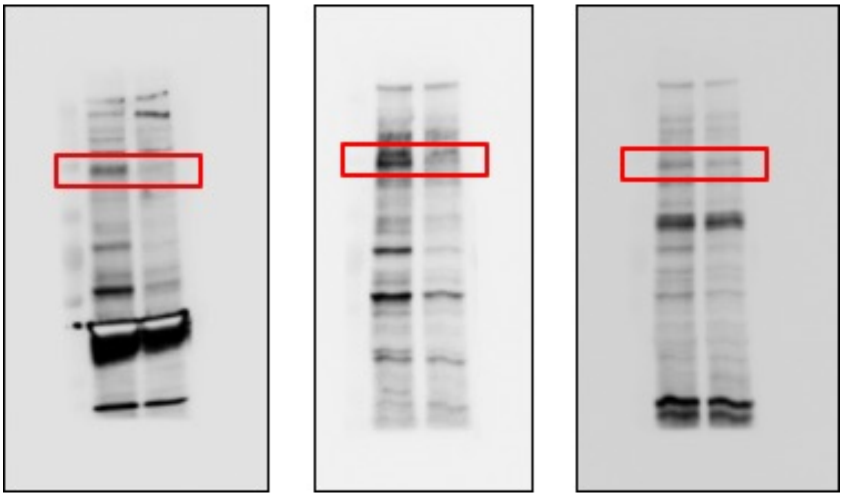

Supplementary Figure 12. Uncropped western blot images. The red sections mark blot and gel results shown in the indicated figures.

Supplementary Table 1. Real-time PCR amplification primers for sequencing (Human).

Species	Gene		Sequence (5' to 3')
**Human**	*RUNX2*	Forward	5'- ACTGGGCCCTTTTTCAGA
		Reverse	5'- GCGGAAGCATTCTGGAA
**Human**	*SP7*	Forward	5'- CGGGACTCAACAACTCT
		Reverse	5'- CGGGACTCAACAACTCT
**Human**	*TNFSF11*	Forward	5'- CAGCACATCAGAGCAGAGAAAGC
		Reverse	5'- CCCCAAAGTATGTTGCATCCTG
**Human**	*ALPL*	Forward	5'- TGGAGCTTCAGAAGCTCAACACCA
		Reverse	5'- TGGAGCTTCAGAAGCTCAACACCA
**Human**	*CTSK*	Forward	5'- ACCGGGGTATTGACTCTGAA
		Reverse	5'- GAGGTCAGGCTTGCATCAAT
**Human**	*NFATC1*	Forward	5'- CTATCCTCTCCAACACCAAA
		Reverse	5'- GTCAGTTTTCGCTTCCATCT
**Human**	*ACP5*	Forward	5'- GATCCTGGGTGCAGACTTCA
		Reverse	5'- GCGCTTGGAGATCTTAGAGT

Supplementary Table 2. Real-time PCR amplification primers for sequencing (Mouse).

Species	Gene		Sequence (5’ to 3’)
**Mouse**	*Itgav*	Forward	5’- ACACTTTGGGCTGTGGAATC
		Reverse	5’- CCACTTAAGAAGCACCTC
**Mouse**	*Mmp3*	Forward	5’- ACCAAGAGAGAGTGTGGATTCTGC
		Reverse	5’- TTGAGAGAGATGGAAACGGGC
**Mouse**	*Mmp13*	Forward	5’- GGTCTTCTGGCACACGCTTTTC
		Reverse	5’- ATGGCATCAAGGGATAGGGCTG
**Mouse**	*Ccr1*	Forward	5’- GTGTTCATCATTGGAGTGGTGG
		Reverse	5’- GGTTGAACAGGTAGATGCTGGTC
**Mouse**	*Ccr2*	Forward	5’- TGTTACCTCAGTTCATCCACGG
		Reverse	5’- CAGAATGGTAATGTGAGCAGGAAG
**Mouse**	*Ccr3*	Forward	5’- TTGCAGGACTGGCAGCATT
		Reverse	5’- CCATAACGAGGAGAGGAAGAGCTA
**Mouse**	*Ccr4*	Forward	5’- TCTACAGCGGCATCTTCTTCAT
		Reverse	5’- CAGTACGTGTGGTTGTGCTCTG
**Mouse**	*Ccr5*	Forward	5’- CATCGATTATGGTATGTCAGCACC
		Reverse	5’- CAGAATGGTAGTGTGAGCAGGAA
**Mouse**	*Ccr6*	Forward	5’- ACTCTTTGTCCTCACCCTACCG
		Reverse	5’- ATCCTGCAGCTCGTATTTCTTG
**Mouse**	*Ccr7*	Forward	5’- CATCAGCATTGACCGCTACGT
		Reverse	5’- GGTACGGATGATAATGAGGTAGCA
**Mouse**	*Ccr8*	Forward	5’- ACGTCACGATGACCGACTACTAC
		Reverse	5’-GAGACCACCTTACACATCGCAG
**Mouse**	*Ccr9*	Forward	5’- CCATTCTTGTAGTGCAGGCTGTT
		Reverse	5’- AAGCTTCAAGCTACCCTCTCTCC
**Mouse**	*Ccr10*	Forward	5’- AGAGCTCTGTTACAAGGCTGATGTC
		Reverse	5’- CAGGTGGTACTTCCTAGATTCCAGC
**Mouse**	*Cx3cr1*	Forward	5’- CCGCCAACTCCATGAACAA
		Reverse	5’- CGTCTGGATGATGCGGAAGTA
**Mouse**	*Ccl1*	Forward	5’- GCTGCCGTGTGGATACAGGA
		Reverse	5’- GAATACCACAGCTGGGGGAT
**Mouse**	*Ccl2*	Forward	5’- GTTCACAGTTGCCGGCTGGA
		Reverse	5’- GACCTTAGGGCAGATGCAGT
**Mouse**	*Ccl3*	Forward	5’- AACATCATGAAGGTCTCCAC
		Reverse	5’- CCAAGACTCTCAGGCATTCA
**Mouse**	*Ccl4*	Forward	5’- ACACCATGAAGCTCTGCGT
		Reverse	5’- CGCTGGAGCTGCTCAGTTC
**Mouse**	*Ccl5*	Forward	5’- CATATGGCTCGGACACCACT
		Reverse	5’- ACACACTTGGCGGTTCCTTC
**Mouse**	*Ccl6*	Forward	5’- AGCGATCGGAGAGTTCAGAGGTG
		Reverse	5’- TTCACCCCAAGAGCCCAGTTTCA
**Mouse**	*Ccl7*	Forward	5’- AGCTACAGAAGGATCACCAG
		Reverse	5’- CACATTCCTACAGACAGCTC
**Mouse**	*Ccl8*	Forward	5’- AGTGCTTCTTTGCCTGCTGCTCATAG
		Reverse	5’- ATGAGAAAACACGCAGCCCAGGCACC
**Mouse**	*Ccl9*	Forward	5’- GATGAAGCCCTTTCATACTGC
		Reverse	5’- GTGGTTGTGAGTTTTGCTCCAATC
**Mouse**	*Cx3cl1*	Forward	5’- CACCTCGGCATGACGAAAT
		Reverse	5’- TTGTCCACCCGCTTCTCAA
**Mouse**	*Gapdh*	Forward	5’- AGTATGACTCCACTCACGGCAA
		Reverse	5’- TCTCGCTCCTGGAAGATGGT
