# Supplementary material for: The HIV co-receptor CCR5 regulates osteoclast function
Source: Nat Commun. 2017 Dec 20;8:2226. doi: 10.1038/s41467-017-02368-5 (PMC5738403; doi:10.1038/s41467-017-02368-5)
Supplement: Supplementary file 3 — Description of Additional Supplementary Files [file 41467_2017_2368_MOESM3_ESM.pdf]

Description of Additional Supplementary Files

File Name: Supplementary Movie 1
Description: Live imaging of wild-type (Ccr5+/+) osteoclasts expressing GFP.

File Name: Supplementary Movie 2
Description: Live imaging of *Ccr5*-deficient (*Ccr5*$^{-/-}$) osteoclasts expressing GFP.

File Name: Supplementary Movie 3
Description: Reconstructed 3D-SIM images of wild-type (*Ccr5*$^{+/+}$) osteoclasts. The cells were subjected to immunofluorescence staining with anti-Vincullin (in purple), anti-Pyk2 (in red) and phalloidin-AlexaFluor 488 (in green).

File Name: Supplementary Movie 4
Description: Reconstructed 3D-SIM images of *Ccr5*-deficient (*Ccr5*$^{-/-}$) osteoclasts. The cells were subjected to immunofluorescence staining with anti-Vincullin (in purple), anti-Pyk2 (in red) and phalloidin-AlexaFluor 488 (in green).

File Name: Supplementary Movie 5
Description: A live cell tracking analysis. Wild-type (*Ccr5*$^{+/+}$) osteoclasts toward the M-CSF gradient was performed using the IMARIS software program.

File Name: Supplementary Movie 6
Description: A live cell tracking analysis. *Ccr5*-deficient (*Ccr5*$^{-/-}$) osteoclasts toward the M-CSF gradient was performed using the IMARIS software program.
